# Functional outcomes following fixation of a marginal distal radius fracture with two commonly used volar locking plates: a retrospective cohort study

**DOI:** 10.1186/s12891-021-04984-1

**Published:** 2022-01-03

**Authors:** Yin-Ming Huang, Chun-Yu Chen, Kai-Cheng Lin, Yih-Wen Tarng, Ching-Yi Liao, Wei-Ning Chang

**Affiliations:** 1 Department of Orthopedics, Kaohsiung Veterans General Hospital, 386 Ta-Chung 1st Road, Kaohsiung City, Taiwan, Republic of China; 2 Department of Occupational Therapy, Shu-Zen Junior College of Medicine and Management, Kaohsiung, Taiwan Republic of China; 3 Department of Biomedical Engineering, I-Shou University, Kaohsiung, Taiwan Republic of China

**Keywords:** Distal radius fracture, Watershed line, Volar locking plate, Flexor tendon irritation, Functional outcome

## Abstract

**Introduction:**

The volar locking plate has been widely used for unstable distal radius fractures to provide early recovery of wrist function. Volar plate prominence to the watershed line has been reported to be related to flexor tendon irritation, and avoid implant prominence in this area was suggested. On the other hand, marginal distal radius fracture patterns required the plate to cross the watershed line, making conflict over plate positioning on marginal distal radius fractures. This study compared functional outcomes in patients with marginal distal radius fractures treated with two different implants.

**Materials and methods:**

A retrospective study was conducted, all patients who received a Synthes 2.4 mm LCP or an Acumed Acu-Loc VLP between January 2015 and December 2018 were reviewed. The marginal distal radius fracture pattern was the most distal horizontal fracture line within 10 mm of the lunate fossa’s joint line. The primary outcomes including patient-reported pain scores, range of motion, and grip strength were assessed. Secondary outcomes included patient-based subjective satisfaction scores of the injured wrist and hand function. The Mayo Wrist Score and the requirement for a secondary procedure related to hardware complications were also recorded.

**Results:**

Forty-two patients met our inclusion criteria. Twenty-one patients were treated with the Synthes 2.4 mm LCP, and 21 patients with the Acumed Acu-Loc VLP. The primary outcome revealed that post-operative range of motion (*P* = 0.016) and grip strengths (*P* = 0.014) were significantly improved in the Acu-Loc VLP group. The MAYO wrist score in the Acu-Loc VLP group was also significantly better (*P* = 0.006).

**Conclusions:**

Despite advances in implant designs, flexor tendon irritation or rupture is still a complication following distal radius’s volar plating. We believe the Acumed Acu-Loc VLP design provided better functional outcomes than the Synthes 2.4 mm LCP if appropriately and carefully placed into its designed-for position. This positioning results in promising patient satisfaction when treating marginal distal radius fractures.

## Introduction

Distal radius fractures are one of the most common skeletal injuries of the wrist, accounting for 14–18% of all fractures in adults [1, 2]. Most distal radius fracture occur in elderly patients, with an increasing incidence in aging populations. Many treatment methods have been described to treat these fractures, including close reduction with casting protection, percutaneous Kirschner wire reduction and fixation, joint-spanning external fixation, and open reduction and internal fixation (ORIF) with volar and (or) dorsal plates [3].

As fracture patterns and mechanisms are widely divergent, few of the available treatment options are applicable to all patients, therefore patient-specific treatments are required [1, 3–5]. In the last decade, surgical techniques and implant designs have advanced considerably. Since its introduction in 2000, the volar locking plate (VLP) has been widely used in patients with unstable distal radius fractures, as it provides secure immobilization, early postoperative mobility, and rapid recovery of wrist function [1, 6–11].

However, distal radius VLP also predisposes the patient to several specific complications. Upon distal radius VLP fixation, overall complications range between 5 and 27%, and include: carpal tunnel syndrome, malunion, intra-articular screw trajectory, mal-positioning of the plate, prominent hardware, possible flexor or extensor tendon irritation, and potential rupture [1, 11–16]. Numerous studies have demonstrated a relationship between flexor tendon irritation (i.e. flexor pollicis longus, (FPL)) or rupture, and volar plate prominence to the watershed line (Soong’s classification grade I or II). These observations suggest surgeons should avoid implant prominence in this area [12–14, 17].

In the past decade, several distal radius VLP mechanisms have been designed to minimize complications, and increase fixation power, thereby leading to promising postoperative outcomes for distal radius fractures [4, 6, 7, 10, 14, 18, 19]. With advanced implant design, surgical technique, and early post-operation rehabilitation, the functional outcome has improved in recent years. The incidence of implant removal rate has decreased from 20% in 1998 to 12% in 2016 [8, 20]. However, not all VLP designs are suitable for marginal distal radius fracture patterns in which fracture line very close to the joint line. Standard VLP designs cannot buttress the fracture fragment at standard positions, which are proximal to the watershed line [17, 18]. Too proximal position of the distal radius VLP, or inadequate selection of the plate in this fracture pattern, can lead to the subsequent displacement of the distal fragment [18]. Accordingly, there is conflict over plate positioning in marginal distal radius fractures, plate positioning the over the watershed line is essential for adequate fixation. Equally, an increased risk of flexor tendon injury is also an issue.

There are only a few previous reports that compare different distal radius VLPs. However, none of these studies focused on VLP designed for marginal distal radius fracture patterns or comminuted intra-articular fractures requiring fixation over marginal fragments [21–23]. In this study, we investigated primary and secondary functional outcomes in patients with marginal distal radius fractures, or comminuted intra-articular fracture patterns, treated with two different distal radius VLP designs: Synthes 2.4 mm LCP™ Distal Radius System Juxta-articular volar plates (Synthes 2.4 LCP) and the Acumed Acu-Loc Wrist Plating System Volar Distal Radius Plate (Acumed Acu-Loc VLP).

## Methods

### Patient selection

We conducted a retrospective study by reviewing all patients who received a Synthes 2.4 mm LCP and an Acumed Acu-Loc VLP. The criteria for using these two implants are the same; the fracture pattern extends beyond the watershed line, and an ordinary distal radius VLP could not secure the fractured fragment. The decision to use the Synthes 2.4 mm LCP or an Acumed Acu-Loc VLP was based on the surgeon’s preference.

**Fig. 1:**
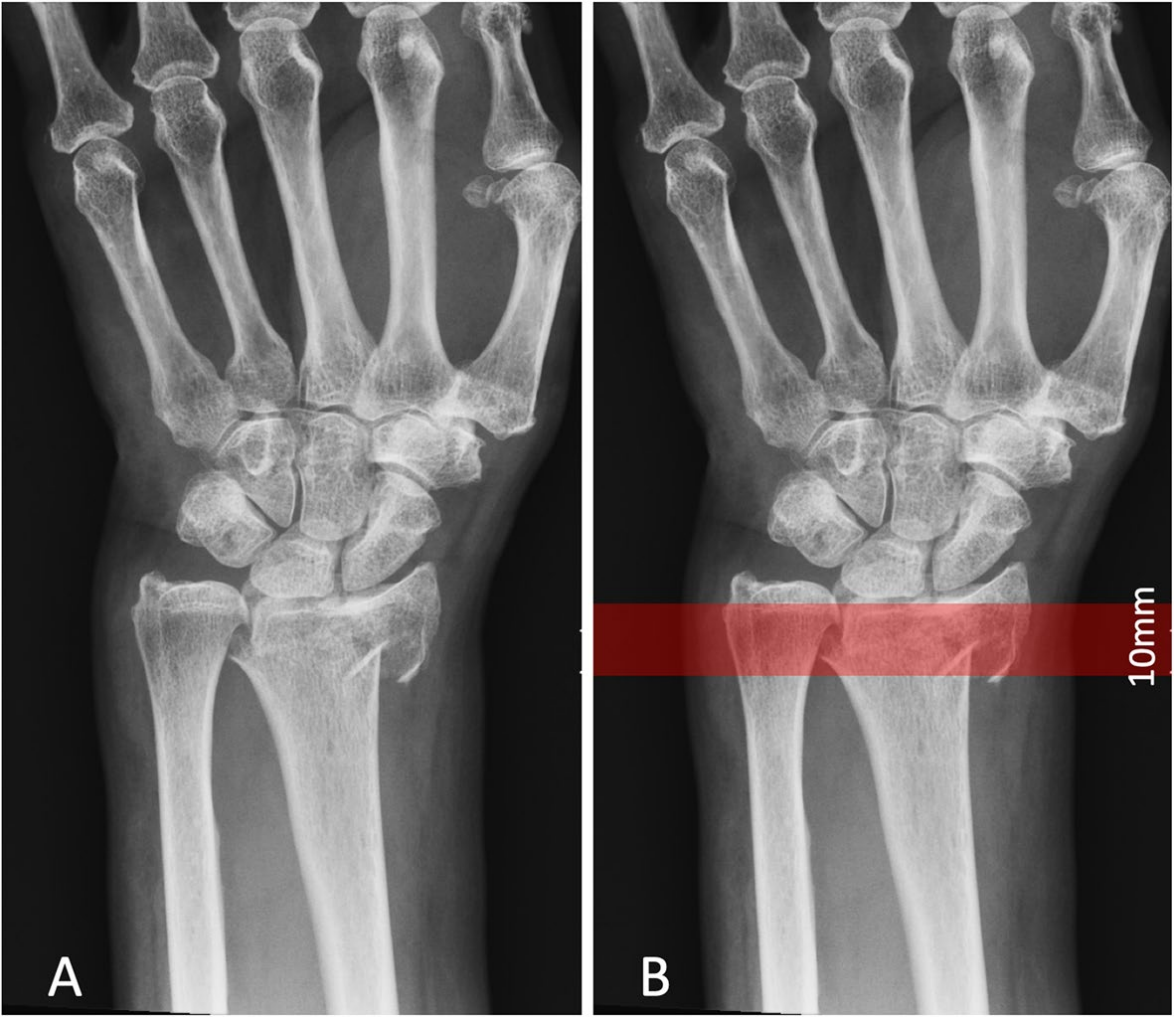
Defining marginal distal radius fracture patterns. A) Pre-operation anteroposterior film of an injured wrist; B) The distal end of a fracture area lies within 10 mm of the joint line of the lunate fossa

Those patients attending between January 2015 and December 2018 were identified. Inclusion criteria: patients receiving distal radius fracture fixation with one of the two study implants, patients with a fracture pattern meet the marginal distal radius fracture definition (Fig. 1), aged > 20 years old when the fracture occurred, and amenable to a minimum follow-up of 24 months. Exclusion criteria: patients with multiple trauma, more than one skeletal fracture, previous injury over the ipsilateral or contralateral wrist, those lost to follow-up within 24 months post-operation, open fracture, and primary injury involving the wrist tendon or neurovascular structure.

### Operation technique and post operation rehabilitation protocol

Hand specialists conducted the operation with a standard, modified Henry approach about 35–45 mm over the volar aspect of the wrist just above the flexor carpi radialis tendon. The Pronator quadratus muscle was cut and elevated for fracture site exposure and later repaired after the plate insertion. The patient was encouraged to begin early finger active ROM with strength immediately after the operation. Meanwhile, the wrist joint was immobilized for ten days with a short arm splint before the first follow-up visit, where the suture was removed. Non-weight bearing and passive wrist ROM was carried on as the patient tolerated after removing the short arm splint. The passive ROM degrees gradually increased until six weeks after surgery, when active ROM was attempted.

### Patient classification

Fracture configuration and patterns were classified radiographically by a senior orthopedic resident and rechecked by a hand surgeon. The definition of a marginal distal radius fracture pattern was assumed to be the most distal horizontal fracture line within 10 mm of the joint line of the lunate fossa, based on anteroposterior (AP) film. Simple volar or dorsal Barton fracture patterns (AO/OTA 2R3B2/3) were excluded (Fig. 1). All pre-operation, intra-operation, and post-operation plan films, and pre-operation computed tomography (CT), intra-operation arthroscopic images (if Arthroscopically assisted reduction and internal fixation was performed) were reviewed and recorded.

### Primary and secondary outcomes

The primary outcomes were: patient reported pain scores (VAS scores) and range of motion and grip strength of the injured wrist when compared to the uninjured wrist at clinical review more than two years after the operation. Patient reported range of motion and grip strength were classified into five groups: 0–24%, 25–49%, 50–74%, 75–99 and 100% of uninjured wrists for each group. Secondary outcomes included patient-based subjective satisfaction scores of the injured wrist, and hand function at work, sports and social activities, and daily life (ranging from 1 to 10: 1 = worst/ unsatisfactory outcome and 10 = best/satisfactory outcome). The X ray of the last follow up more than two years was reviewed and radiological parameters including Soong grade, radial height, radio inclination angle, ulnar variance, articular step-off on AP view, volar tilt angle was recorded. The Mayo Wrist Score and the requirement for a secondary procedure related to hardware complications (i.e., hardware removal, tendon release, tendon irritation, tendon repair due to rupture, loss of reduction or mal-positioning of the plate or screws) were also recorded. All fixation and arthroscope operations were performed by hand surgeons, independent of outcome measurements and statistical analyses.

### Statistical analyses

Data were expressed as the mean ± standard deviation (SD). Comparisons between groups were performed using non-parametric tests. A *P* value < 0.05 was considered statistically significant.

## Results

In the four year interval (2015–2018), 57 patients met our definition of marginal distal radius fracture, 29 patients treat with Synthes 2.4 mm LCP with eight patients excluded in further analysis(1 patient is a foreign patient and cannot understand Chinese nor English, three patients treated initially with dual-plating technique, three patients loss follow up within 24 months, one patient cannot report functional outcome due to dementia); 28 patients treated with Acumed Acu-Loc VLP with seven patients excluded in further analysis(1 patient is a foreigner patient and cannot understand Chinese nor English, one patient treat initially with dual-plating technique, three patients loss follow up within 24 months, one patient suffered from bilateral distal radius fracture at the same time, one patient received radial artery anastomosis with external fixation at initial injury). Total 42 patients met our inclusion criteria. These comprised: 23 females (54.8%) and 19 males (45.2%). None of the fractures were open. Patient ages (at the time of injury) ranged from 23 to 91, with an average age of 51.19 years. Twenty-one patients were treated with the Synthes 2.4 mm LCP, and 21 patients with the Acumed Acu-Loc VLP. Based on fracture types and patterns, 14 patients were extra-articular (AO/OTA 2R3A1-A3; A1:0, A2:13, A3:1) and 28 were complete articular (AO/OTA 2R3C1-C3; C1:13, C2:10, C3:5). The average distance from the most distal horizontal fracture line to the lunate fossa, based on AP film, was 8.33 mm, with a range of 6.2–9.9 mm (Table 1).

**Table 1:** Patient Demographics

		Overall (***N*** = 42)	Plate		P value
			Synthes 2.4 LCP (***N*** = 21)	Acumed Acu-Loc VLP (N = 21)	
Age (Range)		51.2 (23–91)	51.5 (25–84)	50.8(23–91)	0.89*
Gender					0.53**
Male		19	11	8	
Female		23	10	13	
AO fracture type					0.33**
A		14	6	8	
B		0	0	0	
C		28	16	12	
Mean marginal Distance (± SD)		8.2 mm (± 0.94)	8.5 mm (± 0.87)	8.3 mm (± 0.87)	0.35*
Patient request remove Implant		6	4	2	0.66**

*Mann-Whitney U-test; **Fisher exact test

Marginal Distance: Distance from the most distal fracture line to lunate fossa on pre-operation AP plan film.

In terms of combined injuries, ulnar styloid avulsion fractures were documented in 11 patients, but only one patient was treated with a tension band wire. DRUJ stability was checked intra-operatively by the hand surgeon who performed the volar plating. Two patients required DRUJ pinning. One patient with a TFCC traumatic tear was noted during arthroscopic assist reduction and fixation, and was treated with a suture anchor. Die-punch fractures were observed in four patients at pre-operative X-ray and CT.

We observed no significant differences in gender, age when injured, fracture patterns, distance from the most distal horizontal fracture line to the lunate fossa, and concomitant injuries, including DRUJ instability or TFCC injury.

During follow-up, six patients underwent an operation to remove the implant due to hardware-induced discomfort (irritation): four came from the Synthes 2.4 mm LCP group(19.0% of the Synthes 2.4 mm LCP group), and two came from the Acumed Acu-Loc VLP group(9.5% of the Acumed Acu-Loc VLP group). No FPL tendon or other flexor/extensor tendon ruptures were observed in the groups. Similarly, no surgical site infections, loss of reduction, malunion or other complications requiring a secondary intervention were observed.

Primary outcome evaluations revealed that post-operative range of motion (*P* = 0.016) and grip strengths (*P* = 0.014) were significantly improved in the Acumed Acu-Loc VLP group. In this group, 12 of 21 patients (57.1%) recovered 100% range of motion at 24 months post-operation, when compared to 4 of 21 patient (19.0%) in the Synthes 2.4 mm LCP group. In this latter group, 17 of 21 (81.0%) patients attained 75–99% range of motion. In terms of post-operative gripping power, 14 of 21 (66.7%) patients in the Acumed Acu-Loc VLP group recovered 100% power, at 24 months post-operation, when compared to 5 of 21 (23.8%) patients in the Synthes 2.4 mm LCP group. Better post-operative VAS pain scores (1.05 versus 1.33, respectively) were observed in the Acumed Acu-Loc VLP group, when compared with the Synthes 2.4 mm LCP group, however, these data were not statistically significant.

Secondary outcome evaluations of patient based subjective satisfaction indices indicated slightly improved satisfaction in post-operative social activities (9.38 versus 9.19, respectively) and daily activities (9.10 versus 9.05, respectively) in the Synthes 2.4 mm LCP group, when compared with the Acumed Acu-Loc VLP group. In this latter group, post-operative occupation activity satisfaction was 8.57 versus 8.43, and overall satisfaction was 8.86 versus 8.62, respectively when compared with the Synthes 2.4 mm LCP group. However, no statistically significant differences were observed (Table 2).

**Table 2:** Functional Outcome Between Groups

		Overall (N = 42)	Plate		P value
			Synthes 2.4 LCP (N = 21)	Acumed Acu-Loc VLP (***N*** = 21)	
Range of motion					0.016*
0–24%		0	0	0	
25–49%		0	0	0	
50–74%		1	0	1	
75–99%		25	17	8	
100%		16	4	12	
Gripping power					0.014*
0–24%		0	0	0	
25–49%		0	0	0	
50–74%		4	2	2	
75–99%		19	14	5	
100%		19	5	14	
Pain VAS score (± SD)		1.19 (± 1.33)	1.33 (± 1.46)	1.05 (± 1.20)	0.49**
Subjective Functional Score					
Occupation (± SD)		8.50 (± 3.43)	8.43 (± 1.29)	8.57 (± 1.08)	0.698**
Social activity (± SD)		9.29 (± 0.83)	9.38 (± 0.81)	9.19 (± 0.87)	0.466**
Daily activity (± SD)		9.07 (± 1.05)	9.10 (± 1.09)	9.05 (± 1.02)	0.885**
Overall (± SD)		8.74 (± 1.08)	8.62 (± 1.07)	8.86 (± 1.10)	0.483**
MAYO score (± SD)		74.35 (± 25.68)	76.67 (± 8.99)	86.19 (± 12.03)	0.006**

* Fisher exact test; ** Mann-Whitney U-test

Post-operatively, the MAYO wrist score in the Acumed Acu-Loc VLP group was significantly higher than the Synthes 2.4 mm LCP group (86.19 versus 76.67, P = 0.006). We observed no statistically significant differences in terms of patients requiring implant removal. Moreover, we observed no tendon rupture and other complications requiring secondary operations, between groups.

Bone union was achieved in all 42 patients at last follow-up more than two years postoperatively. Due to the marginal distal radius fracture pattern, all of the patients in our study were classified as Soong grade II. The radiological parameter reveals no significant difference in radial height, radial inclination angle, ulnar variance, and volar tilt angle between groups. 2 of 21 patients in the Synthes 2.4 mm LCP group had an articular step-off more than 2 mm compared with 1 of 21 patients in the Acumed Acu-Loc VLP group, 3 of 21 and 4 of 21 patients had an articular step-off between 1 to 2 mm in Synthes 2.4 mm LCP and Acumed Acu-Loc VLP group respectively, no statistically significant difference observed. (Table 3).

**Table 3:** Radiological Outcome Between Groups

		Overall (N = 42)	Plate		P value
			Synthes 2.4 LCP (N = 21)	Acumed Acu-Loc VLP (N = 21)	
Radiological parameters					
Radial Height (± SD)		9.48 mm (±1.40)	9.41 mm (±1.60)	9.54 mm (±1.20)	0.76*
Radial Inclination Angle (± SD)		18.64° (±3.68)	19.09° (±4.02)	18.21° (±3.36)	0.44*
Ulnar Variance (± SD)		0.01 mm (±1.96)	−0.01 mm (±2.13)	0.04 mm (±1.82)	0.92*
Articular step-off (AP view)					0.79**
< 1 mm		32	16	16	
1-2 mm		8	3	4	
> 2 mm		2	2	1	
Volar Tilt Angle (± SD)		5.21° (±5.46)	4.75° (±5.32)	5.67° (±5.69)	0.59*

* Fisher exact test; ** Mann-Whitney U-test

## Discussion

In this study, we compared two different distal radius VLP designs in patients with marginal distal radius fractures, defined as the most distal horizontal fracture line lying within 10 mm of the lunate fossa. Both VLP are widely used volar plating systems designed for fracture very close to the joint, while the fracture pattern required more distal placement of the plate, namely, to cross the watershed line.

The watershed line concept was proposed by Orbay in 2005, and was defined as “the transverse ridge that limits the concave surface of the volar radius”, and was further refined by Nelson and Orbay, as” the theoretical line marking the most volar aspect of the volar margin of the radius” [24, 25] Thus, there is no generally accepted definition of the watershed line, however, other interpretations include: “The distal radial physeal scar”, “The distal border of the pronator quadratus muscle” and “The origin of the volar carpal ligaments” [26].

In spite of these disparate definitions, the watershed line has been widely used as a distal reference point for distal radius volar plating positioning to avoid flexor tendon irritation, tenosynovitis and rupture [1, 10, 11, 13, 15, 17]. Soong et al. defined the watershed line as” the most prominent part of the volar surface of the distal part of the radius, where the flexor tendon lies closest to the plate and bone” [6]. In 2011, these authors reported the clinical relevance of the watershed line with flexor tendon complications, by introducing “Soong Grading” of the plate position of distal radius volar plating. By placing the distal radius VLP close to or across the watershed line, this resulted in Soong Grade I or II positioning on post-operation lateral views, and was related to a higher incidence of flexor tendon complications, such as tendon irritation, fraying and rupture [6, 12, 15, 17] Drobetz and Kustscha-Lissberg et al. reported FPL rupture in 12% of patients after volar plating with an early designed implant [27]. Mehrzad et al. also reported that seven of their 60 patients (12%) with implant- related complications, required secondary surgical interventions [1]. Even with modern low-profile anatomical implant designs, flexor tendon injury incidences of up to 2% have been recorded [6, 10–12, 28, 29].

To avoid or limit complications, Soong et al. recommended that surgeons avoid implant prominence at the watershed line, however, plate positioning during surgery must account for patient anatomy, fracture patterns and implant design, as well as fitting into the contours of the volar distal radius [4, 15, 16]. Attempts to maintain the VLP proximal to the watershed line, and not protrude over the most prominent part of the volar surface on the lateral view (Soong grade 0) are not always achievable [14, 17, 18].

When treating patients with marginal distal radius fracture patterns, placing the VLP distal to the watershed line is inevitable, and makes plate positioning a challenge during operations. Several implants designs have been designed precisely for these scenarios, i.e., the distal edge of the plate is polished, beveled and contoured, or a notch is placed over the trajectory of the FPL to avoid irritation and reduce pressure beneath the FPL tendon [4, 6, 14, 16, 18, 19].

**Fig. 2:**
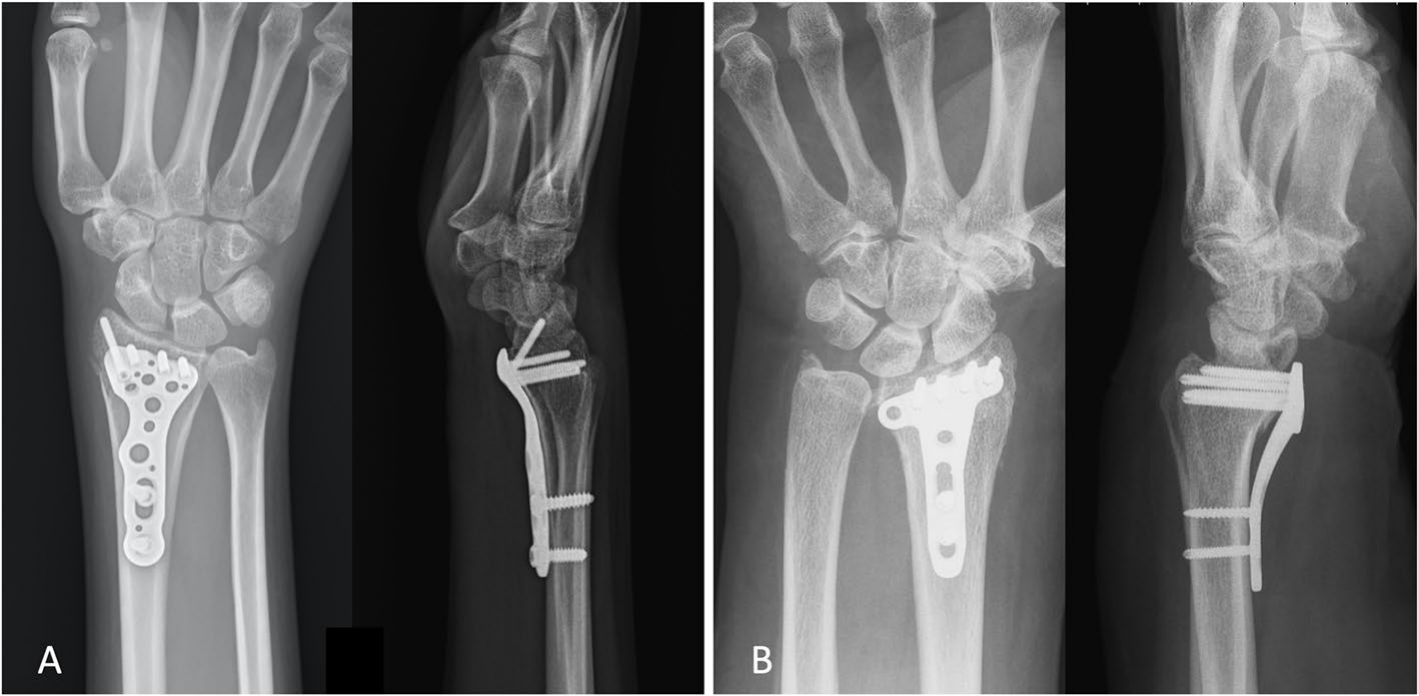
A post-operative anteroposterior and lateral view of an injured wrist. A) The Acumed Acu-Loc VLP group; the implant fits the volar cortex in the lateral view; B) The Synthes 2.4 mm LCP group, note the gap between the implant and the volar cortex

Both study implants were designed for far distal or intra-articular fractures of the distal radius, and to sit distal to the watershed line, but both have with different solution approaches to avoid tendon irritation [30, 31]. The Synthes 2.4 mm LCP™ Distal Radius System Juxta-articular volar plate is pre-contoured to fit the volar cortex of the distal radius. The low plate-and-screw profile, round plate edges and undercut of the plate-head facilitates intraoperative contouring of the plate, based on individual patient anatomy and fracture patterns (Fig. 2) [30].

In contrast, the Acumed Acu-Loc Wrist Plating System Volar Distal Radius Plate has a more rigid and complex pre-contoured design of the distal edge, and is based on the modern module of general population distal radius anatomy. It also comes with a round plate edge, low profile and beveled design [30, 31]]. It has been suggested this design ideally fits the watershed line (cadaver study), whereas the manufacturer states it is “designed to be placed more distal then many other volar plates” (Fig. 2) [31, 32].

In previous studies, the incidence of FPL iatrogenic injury after Acumed Acu-Loc VLP use has been widely reported [6, 12, 13, 17]. A common concern is the flange design of the plate extended toward the radial styloid, even when shaved to a thinned edge, the design is believed to be related to flexor pollicis longus tendon complications [12]. Several newly designed volar locking plates have taken the FPL iatrogenic injury into consideration. The Medartis® APTUS® FPL plate designed an indentation notch over the distal edge of the volar locking plate to reduce the contact between the plate and the FPL tendon [4]. These designs have been proven to be effective by Schilckum et al. using computed tomography combined with high-resolution sonography [10, 19]. Fragment-specific fixation concepts have also been introduced into distal radius fracture treatment. However, a recent study reported that fragment-specific fixation could provide promising outcomes but increase the complication rate [23]. A precise evaluation and computed tomography can help for a more efficient and specific implant selection [4, 16].

**Fig. 3:**
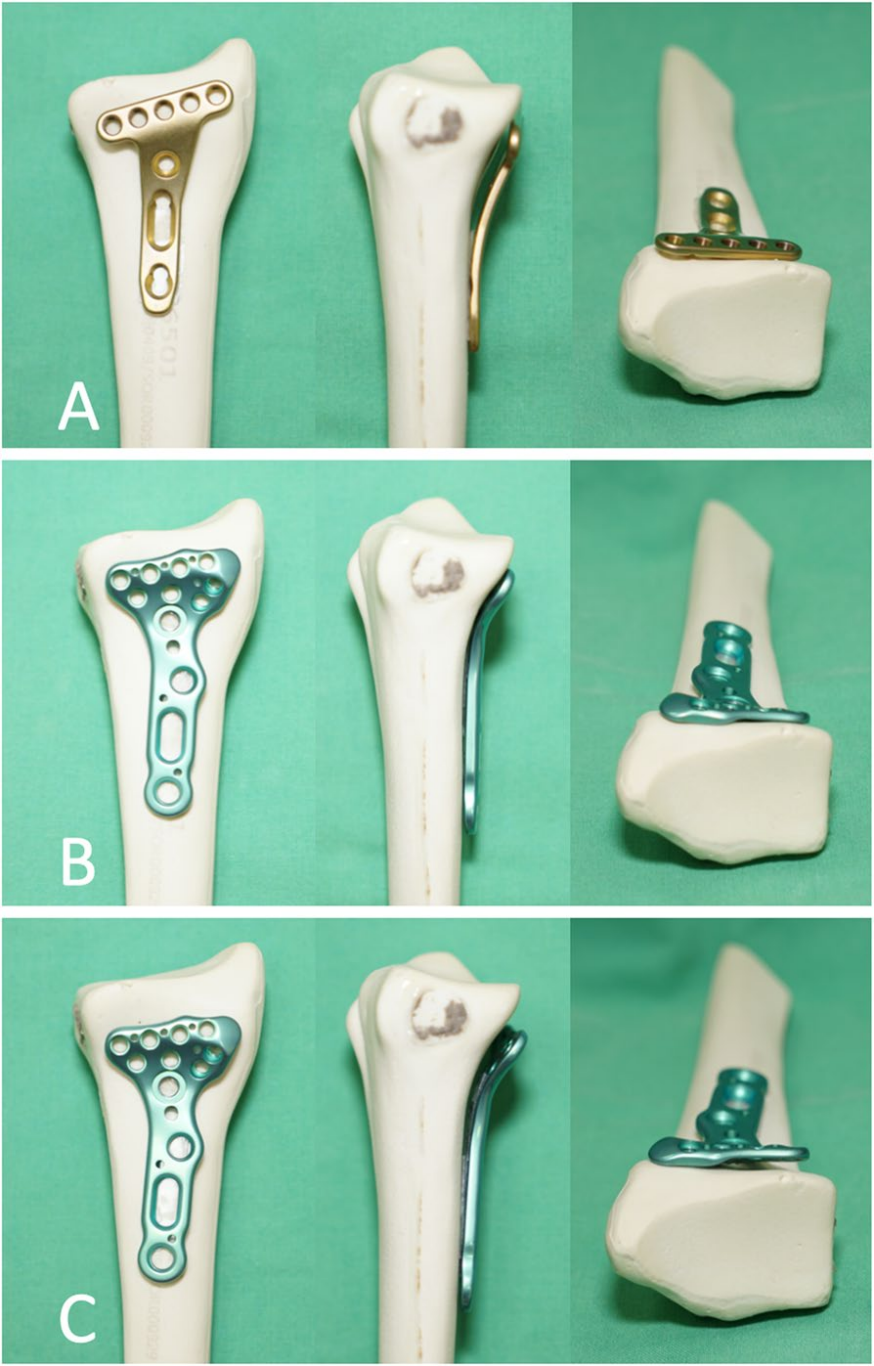
A bone model of anteroposterior, lateral and axial views. A) The Synthes 2.4 mm LCP group, note the plate protrusion on the lateral and axial view; B) The Acumed Acu-Loc VLP group with designed position; C) The Acumed Acu-Loc VLP group with a more proximal position, note the plate protrusion due to plate mis-positioning

In our study, we observed no FPL tear complications. The Acumed Acu-Loc VLP patient group reported better grip power, improved range of motion and Mayo wrist scores. These data indicated that with good Acumed Acu-Loc VLP positioning, patient range of motion was not limited, and plate related tendon irritation become asymptomatic. Both designs made it easier for patient to recover gripping powers and improve wrist functions. We also demonstrated plate position and implant prominence in a distal radius bone model (Fig. 3). When compared with the Synthes 2.4 mm LCP, the Acumed Acu-Loc VLP was a better fit to the volar cortex of the distal radius, and was less prominent on the lateral view. Similarly, the flange concern over the radial styloid was less prominent. This may reduce the risk of flexor tendon complications, resulting in better post-operative functional outcomes.

Furthermore, our bone model (Fig. 3) also indicated if the Acumed Acu-Loc VLP was not placed in its designed-for position, even with a more proximal position not crossing or sitting on the watershed line, this would result in increased prominence. Thus, if the Acumed Acu-Loc VLP is to be used, the surgeon should put the locking plate on the watershed line, even if the fracture pattern does not require marginal fragment fixation.

As the Acumed Acu-Loc VLP is designed to be placed distal to the watershed line, flexor tendon complications are potential risks, post-operation. By carefully placing the Acumed Acu-Loc VLP in its designed-for position, these risks can be reduced, resulting in improved functional outcomes.

However, recent studies have reported correlations between the risk of flexor tendon irritation and implant position that crosses the watershed line and implant prominence to the volar rim of distal radius on lateral view [6, 13, 14, 17, 18]. Thus, we suggest if the fracture pattern of the distal radius does not require marginal fragment fixation, the implant that design to stay proximal to the watershed line should be chosen to maximally reduce complications. However, if placing the plate distal then watershed line is inevitable, such as the marginal distal radius fracture pattern in this study. In choosing the Acumed Acu-Loc VLP and carefully fitting it to the anatomy of the distal radius, we believe this generates better outcomes when compared with the Synthes 2.4 mm LCP.

### Study limitations

Our study had several limitations. It was retrospective in nature, therefore patients were not randomized, and hand-surgeon implant prevalence and operational techniques were not be standardized. The primary outcomes were based on patient report outcomes (PROs), and potentially limit objectivity, as patient expectations and compliance may have influenced the outcomes, besides implant selection. Patient numbers were relatively low, however we must also account for the relatively low incidence of marginal distal radius fracture patterns, therefore we believe our cohort size was appropriate and acceptable. The minimal follow-up period was 24 months post-operation, and was considered adequate in capturing bone healing indices, however some delayed complications may not have been fully ascertained. Asadollahi et al. reported that delayed flexor tendon rupture could occur anywhere between 4 and 68 months, post-operation [33].

## Conclusions

Despite advances in implant designs, flexor tendon irritation or rupture is still a serious complication following distal radius VLP ORIF. Avoid placing the VLP distal than the watershed line and reduced the volar prominence of the implant on lateral view are commonly suggested. But marginal or comminuted intra-articular fracture patterns require more distal fixation, and placing the implant more distal to the watershed line is inevitable. We believe the Acumed Acu-Loc VLP design provided better functional outcomes when compared with the Synthes 2.4 mm LCP, if appropriately and carefully placed into its designed-for position. This positioning results in promising patient satisfaction when treating marginal distal radius fractures.

## Data Availability

The datasets used and/or analysed during the current study are available from the corresponding author on reasonable request.

## Abbreviations

ORIF: open reduction and internal fixation
Synthes 2.4 LCP: Synthes 2.4 mm LCP™ Distal Radius System Juxta-articular volar plates
Acumed Acu-Loc VLP: Acumed Acu-Loc Wrist Plating System Volar Distal Radius Plate
VLP: volar locking plate
AP: anteroposterior
CT: computed tomography
VAS: Visual Analogue Scale
SD: standard deviation
DRUJ: distal radioulnar joint
TFCC: triangular fibrocartilage complex
PROs: patient report outcomes
